# Rare and new occupational inhalant allergens 

**DOI:** 10.5414/ALX01372E

**Published:** 2017-08-04

**Authors:** M. Raulf-Heimsoth, I. Sander, S. Kespohl, V. van Kampen, T. Brüning

**Affiliations:** Department of Prevention and Occupational Medicine, Deutsche Gesetzliche Unfallversicherung, Institute of the Ruhr-University Bochum (IPA), Bochum, Germany

**Keywords:** occupational inhalant allergens, bakers’ allergens, greenhouses, wood dust, insects, fish processing, mites

## Abstract

Occupational airway diseases induced by the inhalation of allergens at workplaces have become common, but the inducing substances are diverse and their pathomechanisms are not always clear. Only few allergens were studied in detail (like wheat flour dust and natural rubber latex) and most of the occupational airway sensitizers were documented only as case reports. In this review rare and exotic occupational Type I-aeroallergens according to their workplace application area (e.g., production of dough and bakery products, handling with decorative and economic plants, wood processing, fish-, shellfish-processing and fish breeding) are described.

German version published in Allergologie, Vol. 34, No. 1/2011, pp. 27-32

## Introduction 

To date far more than 250 workplace substances have been identified to potentially induce Type I allergies. While high-molecular substances usually are proteins or glycoproteins and induce IgE-mediated symptoms like rhinitis, conjunctivitis, bronchial asthma and so forth, in the class of low-molecular substances it is difficult to distiguish between their immunologic-allergic and non-immunologic (chemical irritant/toxic) components. Among the confirmed occupational allergies the most frequent allergy-inducing substances are still flours and flour products, dusts from food and forage as well as from laboratory and farm animals. Only few allergens were studied in detail (like wheat flour dust and natural rubber latex) and most of the occupational airway sensitizers were documented only as case reports. In addition to a comprehensive list and description of occupational inhalant allergens [39] this review presents rather rare and exotic occupational inhalant allergens in different occupational fields and areas of application. 

## Sources of allergen dust in the production of bakery and dough products 

Most studies show that wheat, rye and barley flour are the most important allergens in cases of “baker’s asthma” as an occupational disease [11]. Over the past years new bakery products have been introduced and new or alternative raw materials are being used, resulting in a broader spectrum of allergens for exposed bakers. Case reports show that amaranth [3] or buckwheat [30] as well as lupine flour [4] and soy flour [35] can also act as allergens in workplaces where dough is used (bakeries, production of pizza, pasta and the like). Numerous non-cereal antigens could also be identified as allergens in baker’s asthma. These can either be adjuvants like enzymes or impurities like grain weevils, flour beetles, flour moths [23], cockroaches, storage mites or mold spores of aspergilli and alternaria [11] that can infest the flour due to inadequate storing. 

## Allergen sources in the handling of ornamental and useful plants 

A relatively small occupational group that has so far only rarely been represented in epidemiological studies are gardeners, fruit growers and workers in greenhouses. As, for example, in the Netherlands ornamental and useful plants are intensively cultivated, there are several cross-sectional studies on exposed workers. Greenhouse workers cultivating flowers and harvesting fruits and vegetable can be affected. In cases of pollen allergies the most frequent source for occupational allergies are pollen of flowers and useful plants (fruits and vegetable) that are pollinated by insects. Although chrysanthemum allergy has already been described in several case reports [33], an extensive study in 104 workers in Dutch greenhouses of a chrysanthemum growing company could detect specific IgE against chrysanthemum pollen in 20% of the workers [8]. Also cross-reactivity between mugwort and chrysanthemum pollen was suggested. 

An increased number of allergic symptoms has also been reported for persons with a particularly high exposure when for example pollinating plants. Already in 1990 studies showed allergic symptoms in persons who were responsible for the artificial pollination of cyclamen [38]. Furthermore, allergic diseases like rhinitis, conjunctivitis and bronchial asthma were described for people working with tulips (petals, stems, pollen, bulbs) [33, 38]. Also freesia, gerbera and tuberous begonia led to allergic symptoms in exposed gardeners (pollen extraction, pollination of flowers) [33]. Pollen of Brassica oleracea (cauliflower and broccoli) have been described as new important occupational allergens. In 2006 a study by Hermanides et al. [10] demonstrated that out of 54 cauliflower- or broccoli-exposed workers 44% showed workplace-related symptoms, in 43% the skin prick test was positive and in 26% specific IgE against Brassica oleracea pollen was detectable. Six workers had to give up their job due to these symptoms. Tomatoe pollen (Solanum lycopersicum) and, in particular, also paprika pollen (Capsicum annuum) have been shown to be important occupational allergens in a Dutch cross-sectional study in 472 workers of which 50% suffered from occupation-related symptoms and 35% demonstrated specific IgE against paprika [9]. More recent studies, also coming from the Netherlands, describe an occupation-related allergy induced by strawberry pollen [31]. A partial cross-reactivity with grass and birch pollen could not be excluded. Maize, a wind-pollinated plant species with a low sensitization rate in the general population, lead to a high sensitization rate in laboratory personnel who carried out the manual pollination of maize flowers in greenhouses [28]. 

Idenpendently of pollen sensitization also the occupational contact with other products or parts of plants can lead to sensitization and allergic symptoms. This has been described for green coffee dust (in the manufacturing of green coffee) and castor bean dust (Ricinus communis) [29, 41]. In 2009 Pirson et al. [34] published a case report on rhinoconjunctival and asthmatic symptoms in a patient who had inhaled the dust of Cichorium intybus. The patient was working for an inulin-producing company and thus was exposed to the dust of dried common chicory (inulin is a substance found in some plants that use it as a means of storing energy; the food industry extracts it mainly from asteracea). Diagnostically a positive skin reaction to common chicory and cross-reactions with Bet-v-1 homologues with subsequent oral allergy syndrome to carrot and celery were demonstrated. 

In addition to ornamental and useful plants that represent the main source of exposure in gardeners, fruit growers and greenhouse workers, there are also cases of sensitization and respiratory allergies against spider mites that feed on chlorophyll (e.g., Tetranychus urticae), and also allergies against various species of Mesostigmata (e.g., Amblyseius cucumeris) [7]. The latter are used in greenhouses as a biological pest control for Tetranychus urticae. Spider mites (Tetranychidae) are plant parasites that are found across the world The European red mite (Panonychus ulmi) infests mainly fruit trees and vegetable plants, while the related species Tetranychus urticae can be found on various other sorts of plants. Further well-known species are Panonychus citri and Tetranychus telarius. A review article [19] indicates the prevalence of sensitization to Panonychus citri in 181 lemon farmers to be 16.5%. It was shown that the sensitization to Panonychus citri was the most common reason for asthmatic and rhinitic symptoms in these lemon farmers. Among 725 apple farmers sensitizations against Panonychus ulmi and Tetranychus urticae prevailed. 5% of these farmers showed an isolated skin reaction against spider mites. Although there is a certain cross-reactivity between house dust, storage and spider mites, the occupation-related sensitization to spider mites must be attributed to excessive exposure. As in greenhouses and in the handling of plant material workers can also be exposed to molds which can also be the source of sensitization. 

## Sources of allergen dust in the processing of wood products 

IgE-mediated Type I allergies to woods are rare [18]. The diagnosis of wood dust-related allergic airway diseases is frequently complicated by the lack of allergen extracts; thus, in many cases it is hardly possible to identify the allergen. So far, primarily the plicatic acid of the Western red cedar has been identified as a low molecular weight substance with sensitizing potential, its clinical relevance, however, is controversly discussed [42]. In contrast to many other types of wood, which mainly induce allergic skin reactions, the tropical Abachi wood has a very high protein content that makes it an effective airway sensitizer for Type I reactions. One relevant single allergen could be identified and charcterized [16]. Another allergologically relevant wood is black locust (Robinia pseudoacacia) [15]. This wood is used for example as a substitute for teak and thus is frequently used in outdoor areas. Occupational exposure to black locust wood lead to clinically relevant IgE-mediated sensitizations. The IgE-binding proteins were identified to be 27 kD- and 47 kD-sized molecules. Recently, an occupation-related softwood allergy could be proven and the responsible softwood allergens could be specified and identified [17]. A current meta-analysis on the risk of respiratory diseases caused by exposure to wood dust could show that workers exposed to wood dust had a significantly increased risk to develop respiratory diseases; in addition to the wood dust exposure ethnic and gender-specific factors influenced the risk [32]. 

## Sources of allergen dust in the processing of fish, shellfish and crustaceans as well as in fish farming 

Professions that are exposed to fish, shellfish and crustaceans include fishermen and fish breeders, but also sales persons, storekeepers, carriers, workers in the fish processing industry, catering staff (cooks, waiters) and partially also scientists and laboratory staff. Furthermore, shell grinders and jewelers can be affected [14]. Occupational exposure to fish, shellfish and crustaceans is mainly due to the inhalation of dust, aerosols and vapor that result from cleaning, cutting, cooking or drying the animals [13]. This current review analyzes 33 articles published from 2000 to 2009 regarding occupation-related airway sensitization to fish, shellfish and crustaceans. In the analyzed studies the prevalence of occupation-related asthma was between 2% and 36%. In most studies allergen-specific IgE-antibodies were detected in vitro (IgE testing) and in skin prick testing. In contrast to food allergy no major allergens for airway sensitization have been identified yet. It is suspected that tropomyosin, e.g., as a muscle protein in crustaceans, may play a major role also in airway sensitization, which could also be the reason for cross-reactions with house dust mite. Also the fish parasite Anisakis simplex has been described as an occupationally relevant allergen in the processing of fish [27]. A Portuguese case report describes the simultaneous occurrence of a Type IV (protein contact dermatitis) and a Type I (allergic asthma) reaction to Anisakis in a worker in the fish processing industry [1]. 

Next to fish and shellfish there are also reports of insects that cause occupation-related allergic respiratory diseases. Hemoglobin of chironomid larvae (Chironomidae) could be identified as a potent inhalable allergen in fish breeders and aquarium hobbyists. The structure, immunology and clinical impact of chironomid hemoglobins as potent allergens have been studied very intensively. The best analyzed species is Chironomus thummi, which expresses 12 homologous hemoglobins [21]. Numerous studies demonstrate that on average 20% of exposed persons are sensitized. This applies for the contact with the adult animal as well as with the larva; the latter is relevant from an occupational point of view. In Germany the larvae are frequently used as provender for fish so that workers in fish feed factories, pet shop staff and aquarium hobbyists are exposed [20, 21]. 

## Further occupational allergen sources 

In agriculture respiratory allergies play a major role. In this context allergens can stem from the hair of the cattle or from mites (mostly storage mites) [26]. Exposure to certain allergens, like for example allergens of the housefly (Musca domestica), can lead to IgE-mediated allergic symptoms in individual patients. In a locust breeder, who bred locusts for reptile food, occupational asthma due to the allergen of the migratory locust was detected; the responsible allergen could be identified by mass spectrometry [37]. Enzymes that are industrially isolated from fungal cultures, like for example phytase from Aspergillus niger, can also cause occupational sensitizations [5]. A recent article [22] describes isolated occupational allergy to lipase from Rhizopus oryzae caused by exposure to an enzyme mixture of lipase, amylase and pepsin in the pharmaceutical industry. Another case in the pharmaceutical industry, in the production of tablets, was described by Maniu et al. [24]: sensitization against cornstarch was detected in an apprentice and the authors suggested cornstarch as a potential occupational allergen. 

In the production, packaging and transport of foods there are numerous potential allergen sources that can cause allergic airway diseases [12]. A current review on the role of inhalable food allergens for occupational asthma has been composed by Cartier [4]. This article provides an extensive list of various food components and additives for which cases of allergic asthma following occupational exposure have been described. Further reports on single cases demonstrate that, among others, workers in the production of sausages can be affected from respiratory symptoms. The fungus Penicillium camemberti, which is used for the refinement of this kind of food, is suspected to be the cause for the symptoms. One case of IgE-mediated asthma caused by Penicillium camemberti could be demonstrated in a packer of air-dried peperoni [25]. A current South African article [38] describes the exposure, sensitization and symptoms of three workers in a spice mill who were exposed to high concentrations of the dust of dried spices (chili pepper, garlic and onion powder) for a longer period of time. 

Approximately 140 low-molecular substances that can cause asthma have been described [6]. In some cases specific IgE can also be induced by high-molecular substances. In industrialized countries diisocyanates are one of the main reasons for occupation-related obstructive airway diseases. Acid anhydrates are model substances for allergic occupational asthma caused by low molecular weight substances, as for these substances – in contrast to ioscyanates and numerous other substances – it is possible to prove sensitization in a high percentage of exposed persons showing symptoms [36]. While platinum salts are low molecular weight substances that frequently cause occupational asthma, only one case has been published for iridium [2]. 

Changes in work processes, the introduction of new technologies and the use of new substances can lead to the exposure to new allergens and thus to new sources for sensitization. 

## Conflict of interest 

The authors have no conflicts of interest. 
